# Supplementary material for: Structural Assignment of the Product Ion Generated from a Natural Ciguatoxin-3C Congener, 51-Hydroxyciguatoxin-3C, and Discovery of Distinguishable Signals in Congeners Bearing the 51-Hydroxy Group
Source: Toxins (Basel). 2024 Feb 6;16(2):89. doi: 10.3390/toxins16020089 (PMC10892285; doi:10.3390/toxins16020089)
Supplement: Supplementary file 1 [file toxins-16-00089-s001.zip › toxins-2813151-supplementary.pdf]

toxins

Supplementary materials: Structural Assignment of the Product Ion Generated from a Natural Ciguatoxin-3C Congener, 51-Hydroxycigatoxin-3C, and Discovery of Distinguishable Signals in Congeners Bearing the 51-Hydroxy Group

Formula	Mass	Compound	Description	C	H	D	F	O				
	theoretical value			12.000000	1.007825	2.014102	18.998403	15.994915	$[M-H]^-/[M+H]^+$	*m/z* (calcd)	*m/z* (found)	mass accuracy (ppm)
M C57H82O17	1038.55520			57	82			17	1.007825	1039.5630	1039.5647	1.6
C57H80O16	1020.54464	**B16**		57	80			16	1.007825	1021.5525	1021.5508	-1.6
C57H78O15	1002.53407	**B17**		57	78			15	1.007825	1003.5419	1003.5413	-0.6
C57H76O14	984.52351	**B18**		57	76			14	1.007825	985.5313	985.5294	-2.0
C55H78O15	978.53407	**A9**		55	78			15	1.007825	979.5419	979.5385	-3.5
C57H74O13	966.51294	**B19**		57	74			13	1.007825	967.5208	967.5161	-4.8
C55H76O14	960.52351	**A10**		55	76			14	1.007825	961.5313	961.5296	-1.8
C57H72O12	948.50238	**B20**		57	72			12	1.007825	949.5102	949.5080	-2.3
C55H74O13	942.51294	**A11**		55	74			13	1.007825	943.5208	943.5180	-2.9
C57H70O11	930.49181	**B21**		57	70			11	1.007825	931.4996	931.4953	-4.7
C55H72O12	924.50238	**A12**		55	72			12	1.007825	925.5102	925.5189	9.4
C57H68O10	912.48125	**B22**		57	68			10	1.007825	913.4891	913.4797	-10.3
C57H66O9	906.47656	**A1**		51	70			14	1.007825	907.4844	907.4851	0.8
C51H70O14	894.47068	**B23**		57	66			9	1.007825	895.4785	895.4706	-8.8
C51H68O13	888.46599	**A2**		51	68			13	1.007825	889.4738	889.4670	-7.7
C51H66O12	870.45543	**A3**		51	66			12	1.007825	871.4633	871.4534	-11.3
C51H64O11	852.44486	**A4**		51	64			11	1.007825	853.4527	853.4475	-6.1
C31H40O8	540.27232	**A5**		31	40			8	1.007825	541.2801	541.2794	-1.4
C31H38O7	522.26175	**A6**		31	38			7	1.007825	523.2696	523.2692	-0.7
C28H44O8	508.30362	**B1**		28	44			8	1.007825	509.3114	509.3077	-7.3
C31H36O6	504.25119	**A7**		31	36			6	1.007825	505.2590	505.2612	4.3
C28H42O7	490.29305	**B2**		28	42			7	1.007825	491.3009	491.3017	1.7
C26H40O8	480.27232	**B6**		26	40			8	1.007825	481.2801	481.2808	1.4
C28H40O6	472.28249	**B3**		28	40			6	1.007825	473.2903	473.2848	-11.7
C26H38O7	462.26175	**B7**		26	38			7	1.007825	463.2696	463.2709	2.9
C28H38O5	454.27192	**B4**		28	38			5	1.007825	455.2797	455.2749	-10.7
C26H36O6	444.25119	**B8**		26	36			6	1.007825	445.2590	445.2574	-3.6
C24H38O7	438.26175	**B24**		24	38			7	1.007825	439.2696	439.2684	-2.7
C28H36O4	436.26136	**B5**		28	36			4	1.007825	437.2692	437.2653	-8.9
C26H34O5	426.24062	**B9**		26	34			5	1.007825	427.2484	427.2462	-5.3
C24H36O6	420.25119	**B25**		24	36			6	1.007825	421.2590	421.2626	8.5
C26H32O4	408.23006	**B10**		26	32			4	1.007825	409.2379	409.2342	-9.0
C24H34O5	402.24062	**B26**		24	34			5	1.007825	403.2484	403.2456	-7.1
C26H30O3	390.21949	**B11**		26	30			3	1.007825	391.2273	391.2247	-6.7
C9H14O3	170.09429	**B12**		9	14			3	1.007825	171.1021	171.1038	9.8
C9H12O2	152.08373	**B13**		9	12			2	1.007825	153.0916	153.0920	2.9
C9H12O2	148.08882	**B28**		10	12			1	1.007825	149.0966	149.0961	-3.6
C8H12O2	140.08373	**B14**		8	12			2	1.007825	141.0916	141.0904	-8.2
C8H12O2	134.07316	**B27**		9	10			1	1.007825	135.0810	135.0794	-11.8
C8H10O	122.07316	**B15**		8	10			1	1.007825	123.0810	123.0800	-8.0
C8H10O	114.06808	**B29**		6	10			2	1.007825	115.0759	115.0756	-2.6
C7H8O	108.05751	**A8**		7	8			1	1.007825	109.0653	109.0652	-1.3

Table S1. Assignment of ions contained in product ion spectra from [M+H]+ of 51-hydroxy CTX3C.

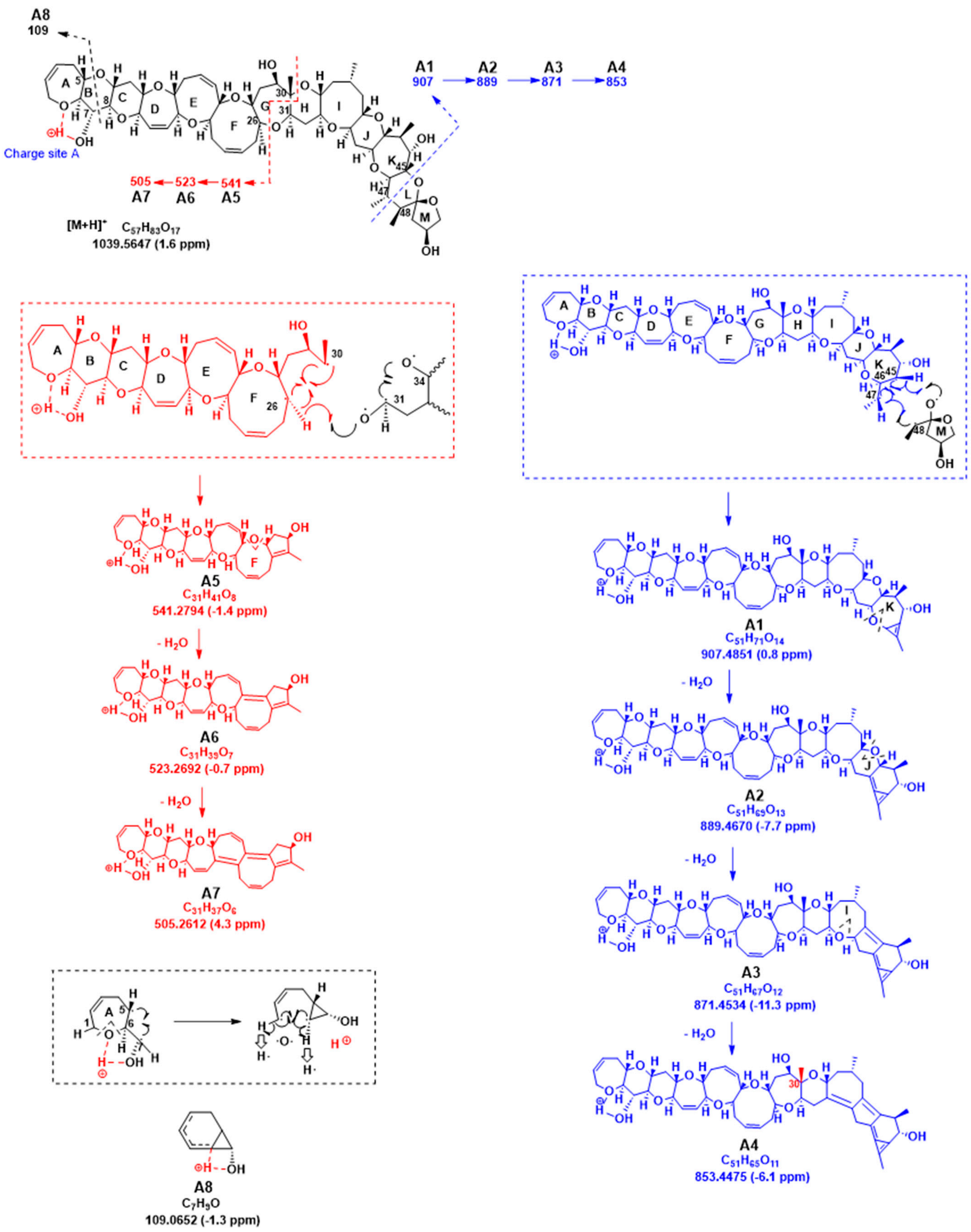

Figure S1. Structures of product ions induced by 51-hydroxy CTX3C at charge site A.

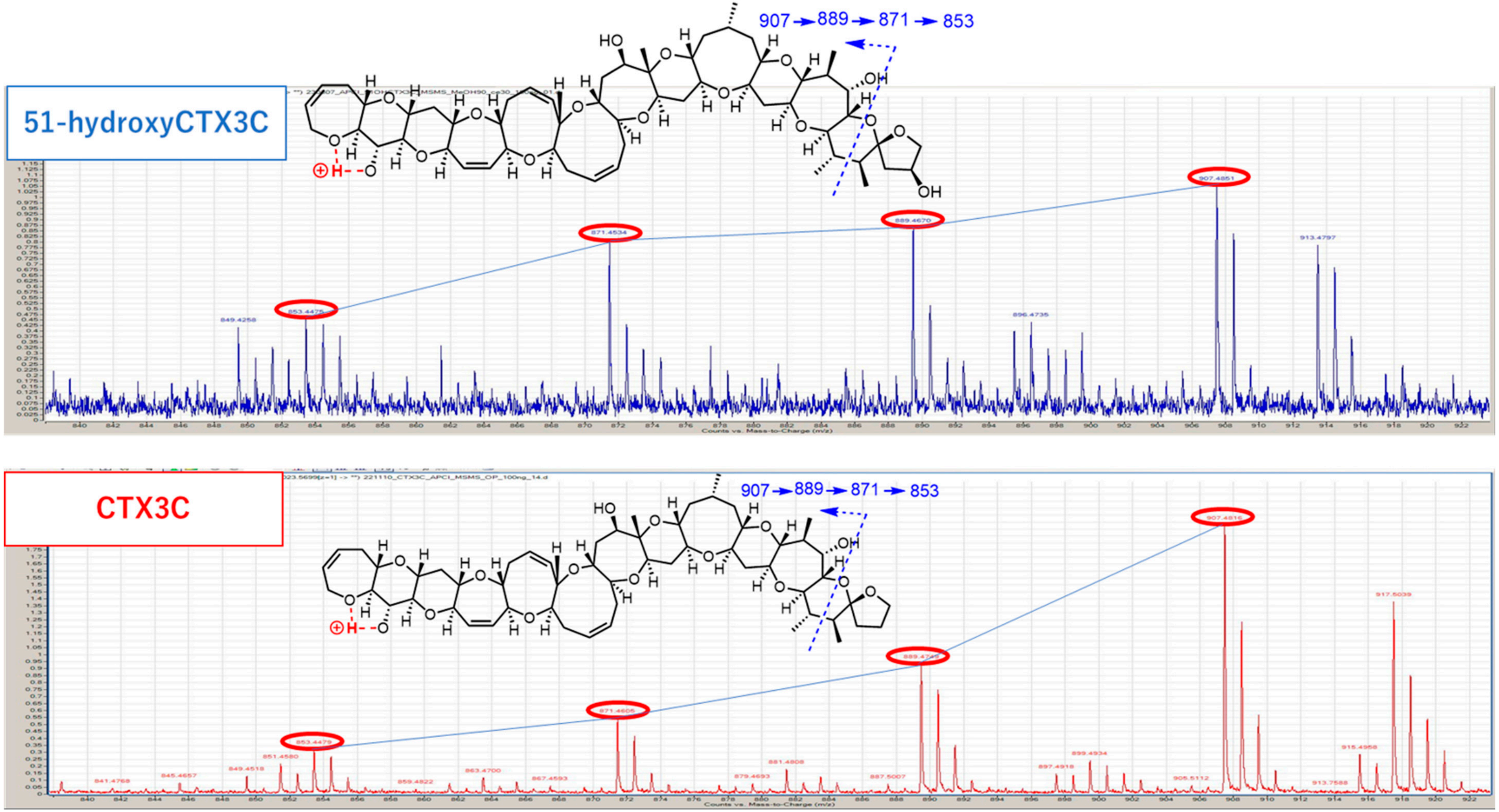

Figure S2. MS/MS product ion spectrum (1) of 51-hydroxy CTX3C [M+H]+ as a precursor ion (A) and CTX3C [M+H]+ as a precursor ion (B).

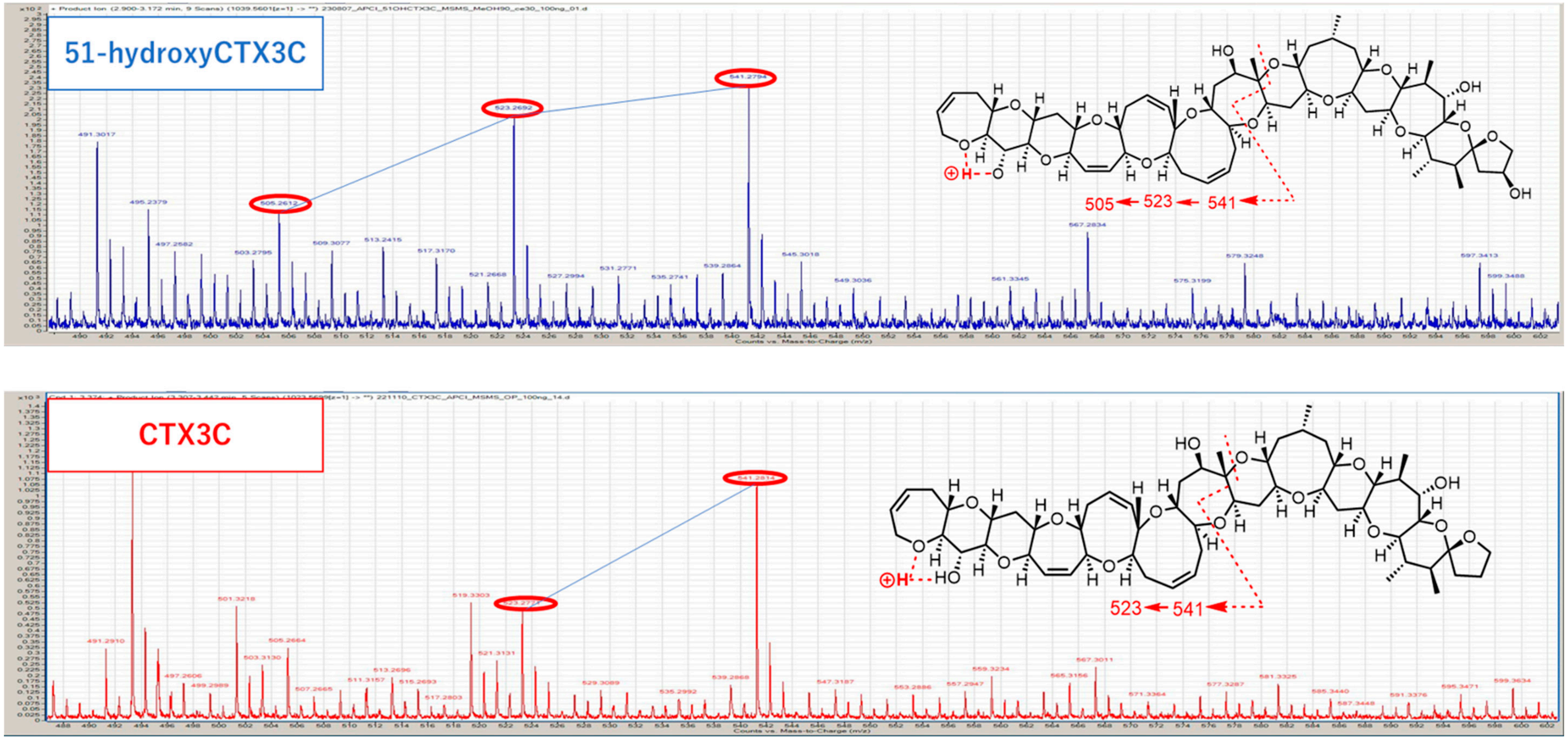

Figure S3. MS/MS product ion spectrum (2) of 51-hydroxy CTX3C [M+H]+ as a precursor ion (A) and CTX3C [M+H]+ as a precursor ion (B).

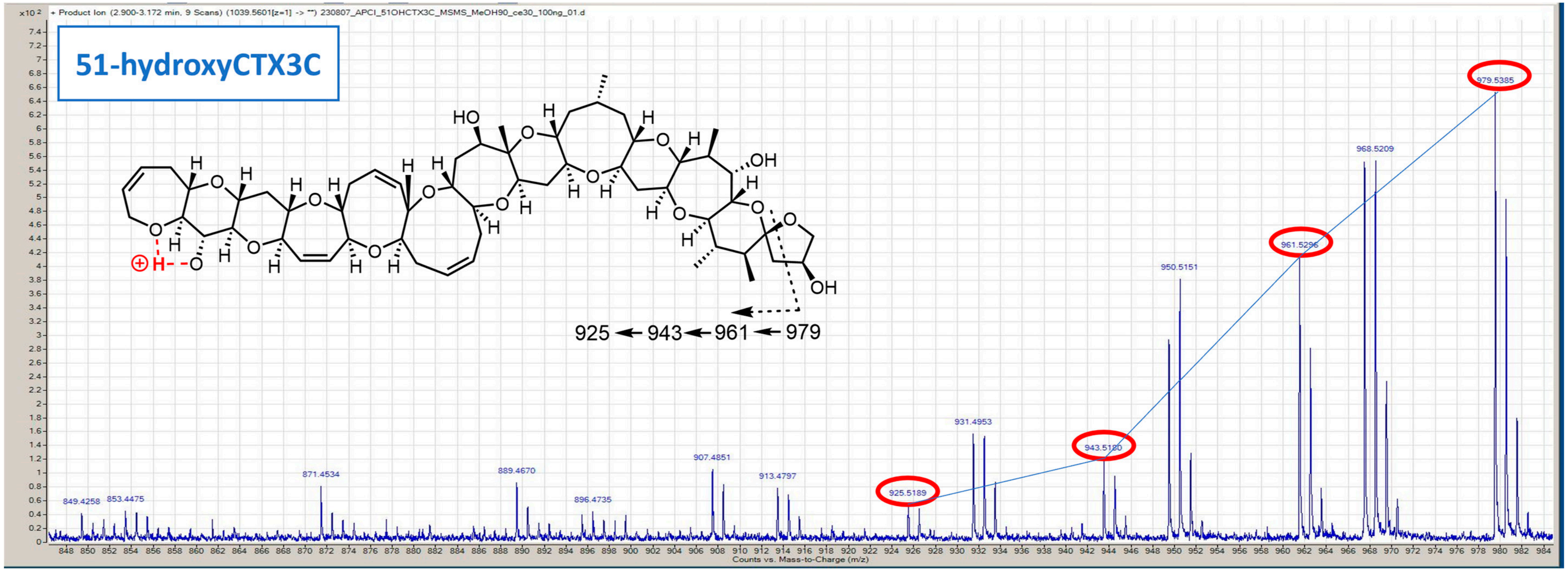

Figure S4. MS/MS product ion spectrum (3) of 51-hydroxy CTX3C [M+H]+ as a precursor ion (m/z 850-980).

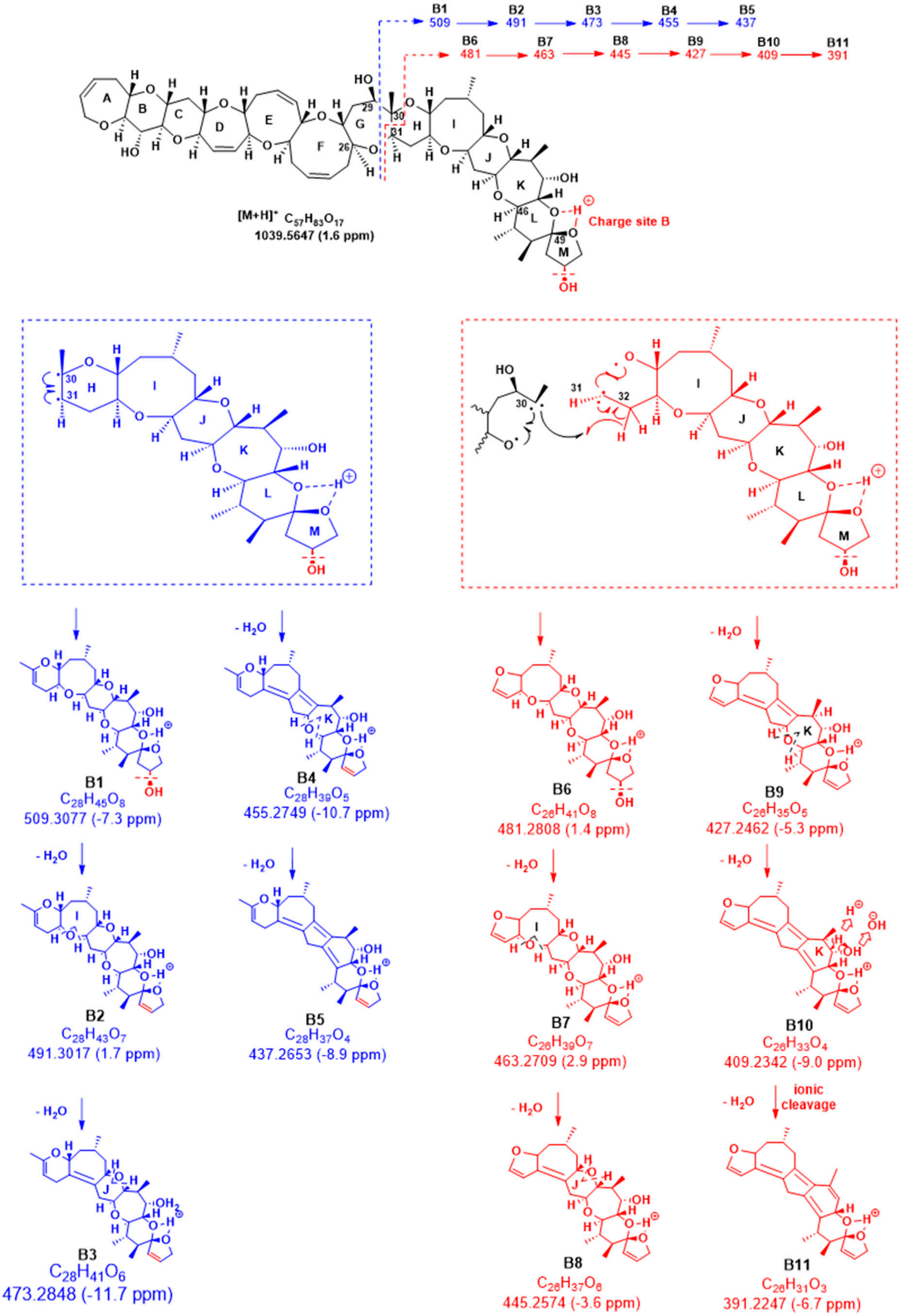

Figure S5. Structures of product ions induced by 51-hydroxy CTX3C at charge site B (1).

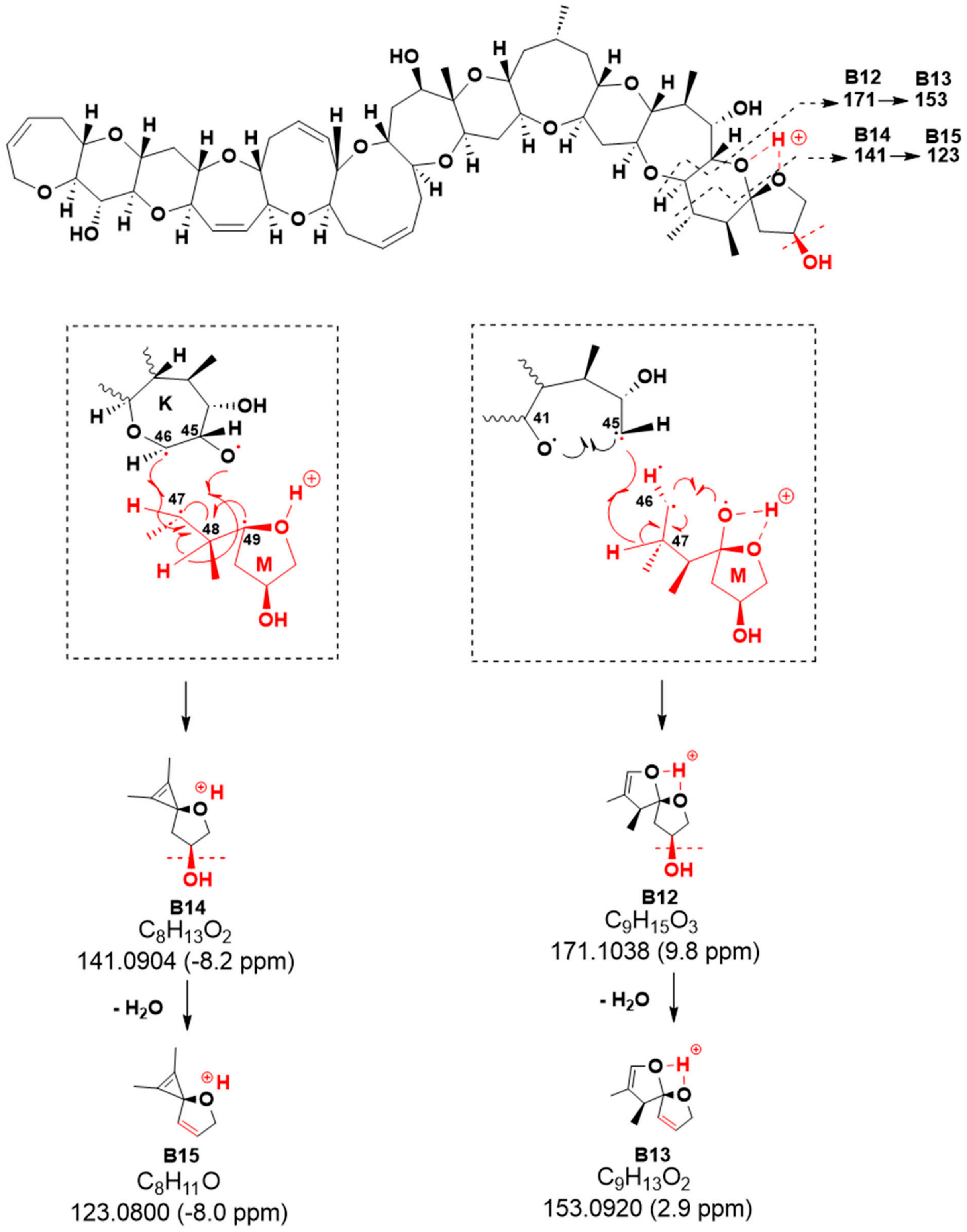

Figure S6. Structures of product ions induced by 51-hydroxy CTX3C at charge site B (2).

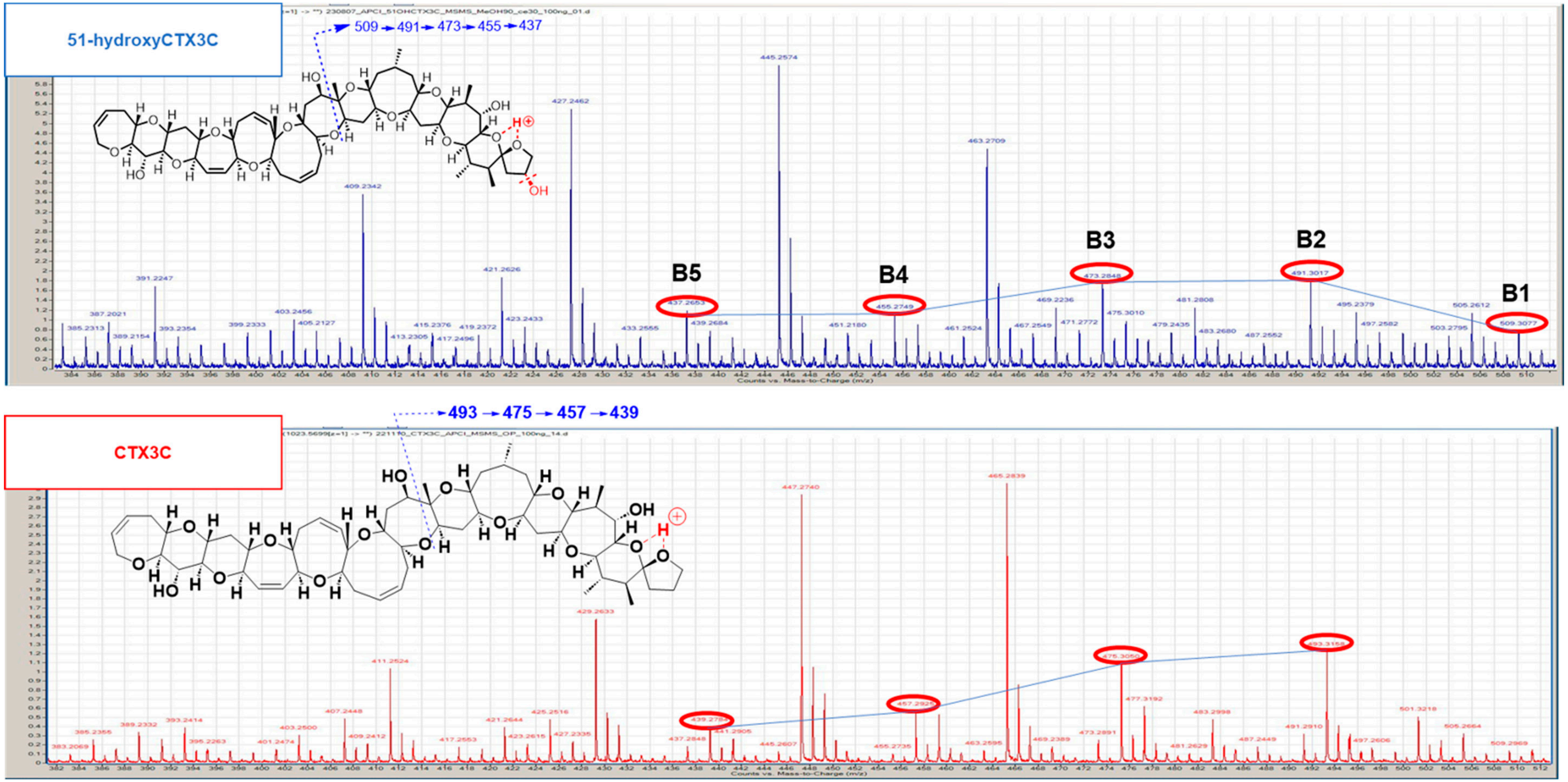

Figure S7. MS/MS product ion spectrum (4) of 51-hydroxy CTX3C $[M+H]^+$ as a precursor ion (A) and CTX3C $[M+H]^+$ as a precursor ion (B).

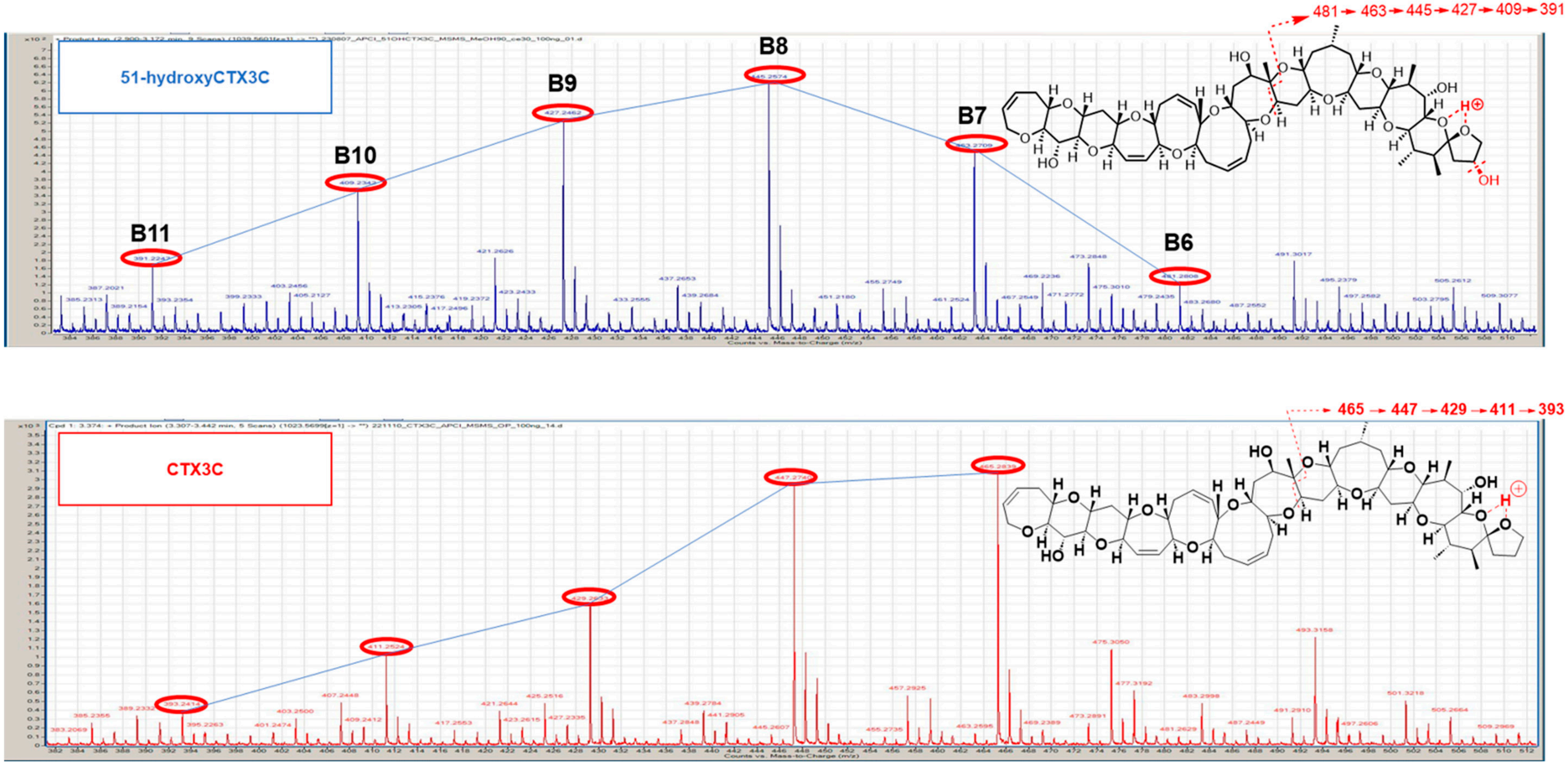

Figure S8. MS/MS product ion spectrum (5) of 51-hydroxy CTX3C [M+H]+ as a precursor ion (A) and CTX3C [M+H]+ as a precursor ion (B).

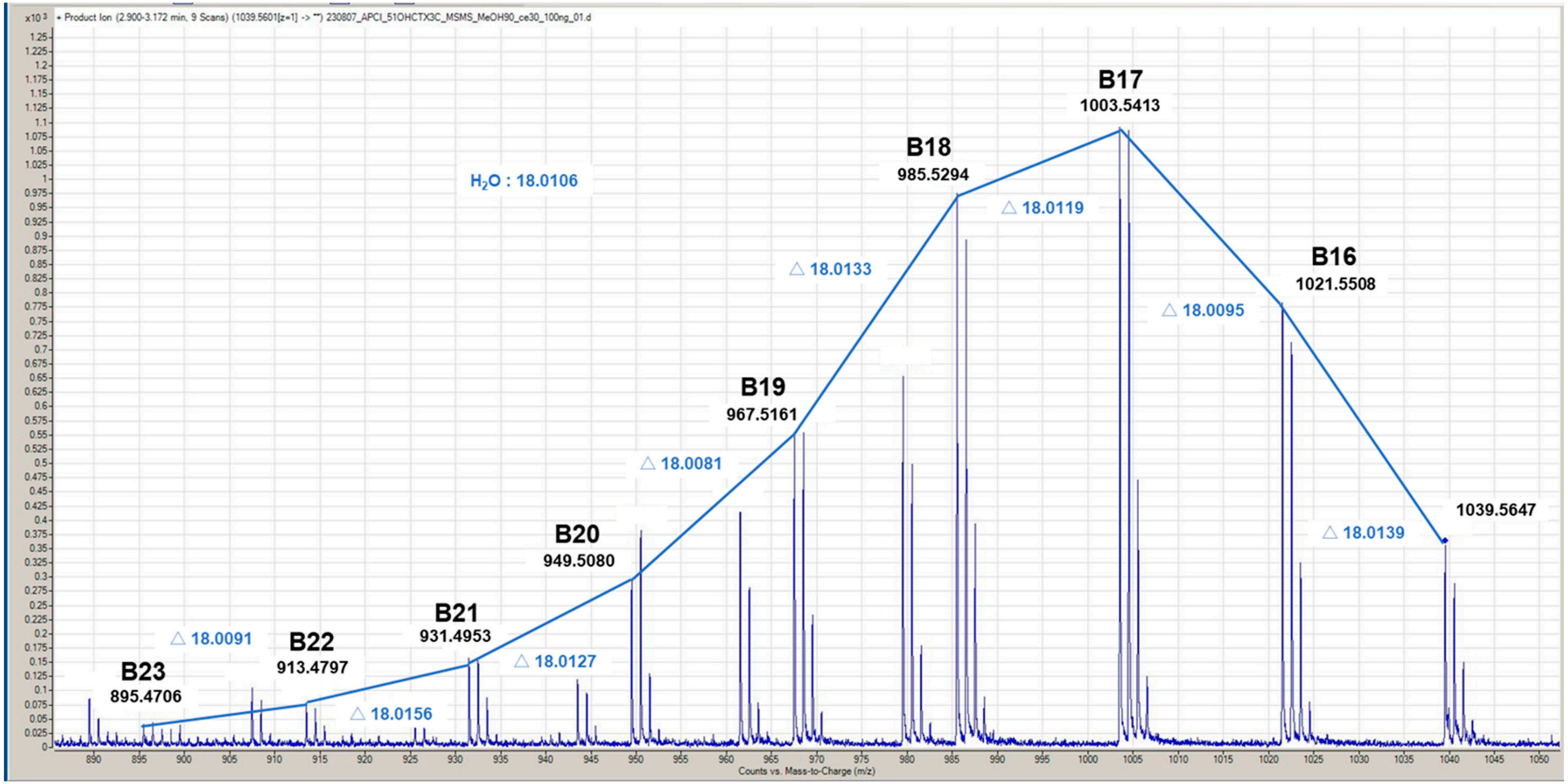

Figure S9. MS/MS product ion spectrum (6) of 51-hydroxy CTX3C [M+H]+ as a precursor ion (m/z 890-1050).

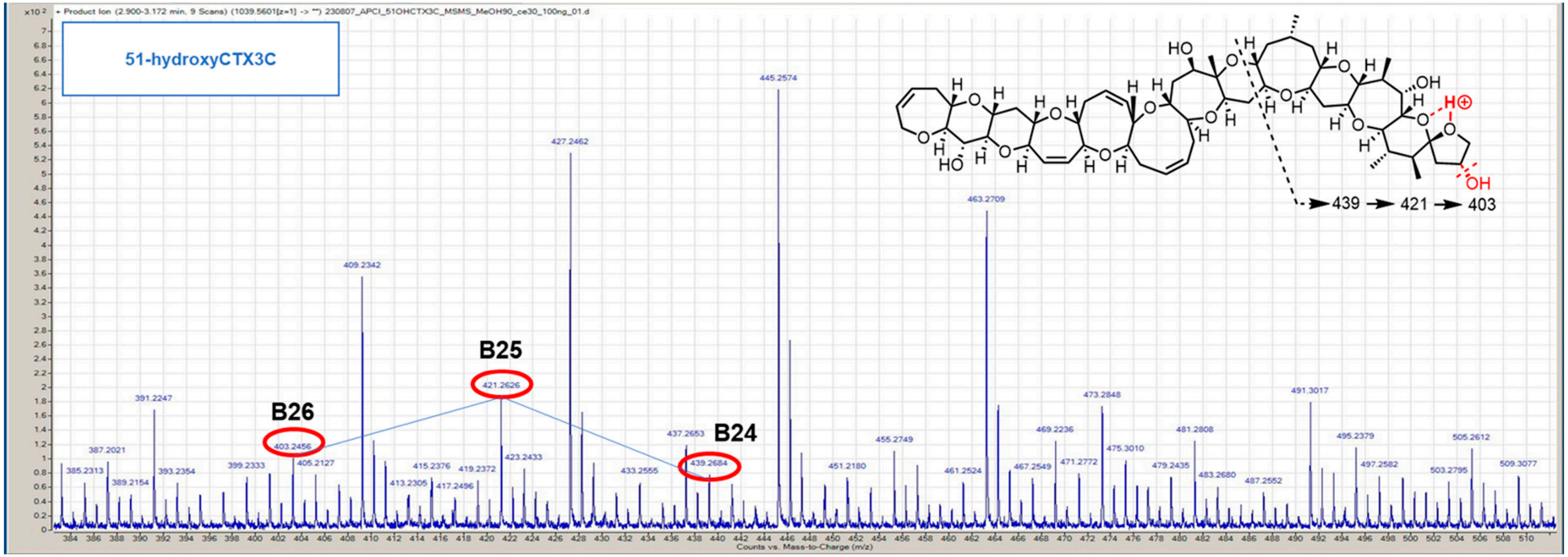

Figure S10. MS/MS product ion spectrum (7) of 51-hydroxy CTX3C [M+H]+ as a precursor ion.

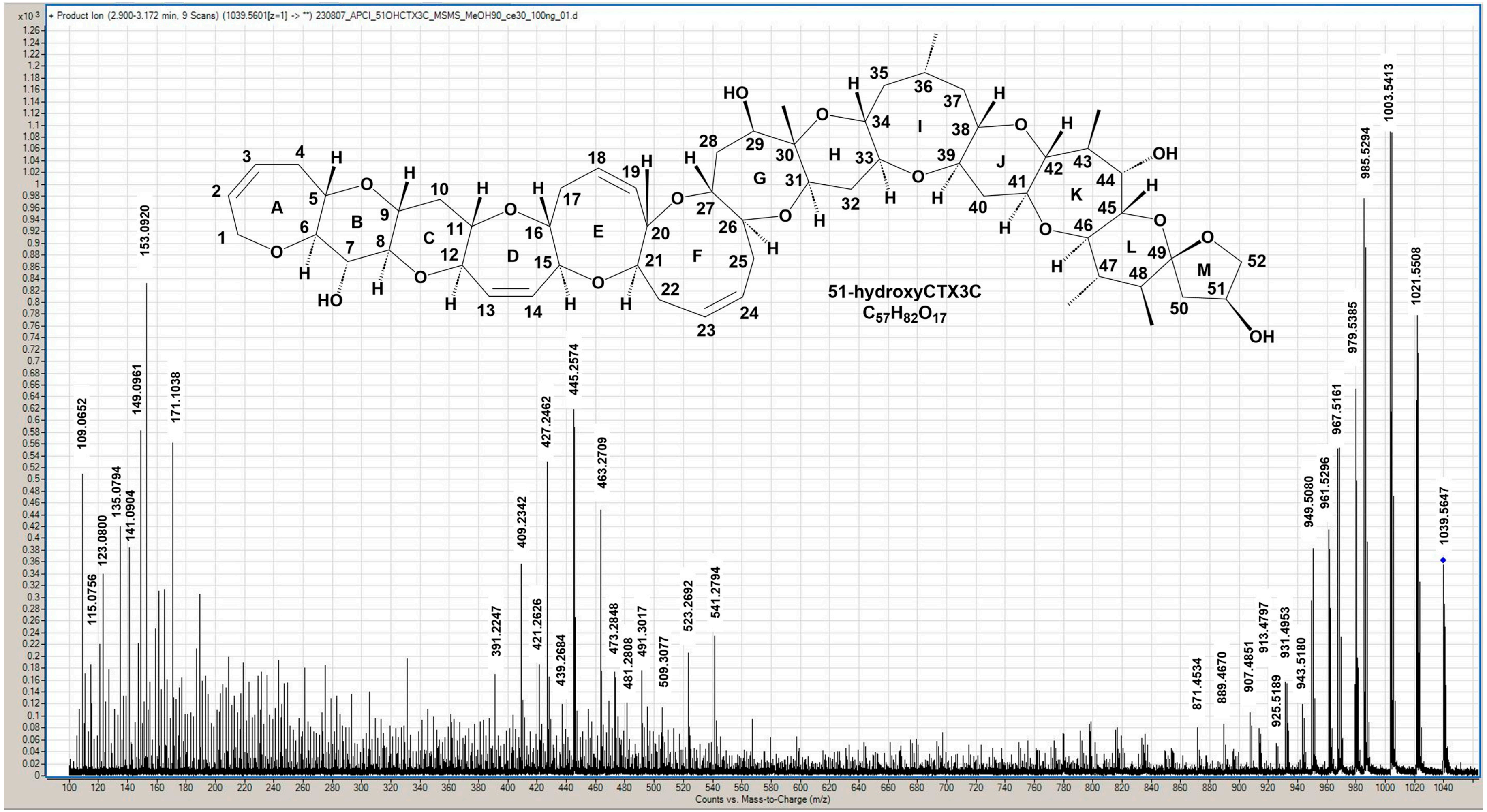

Figure S11. APCI-MS/MS product ion spectrum of 51-hydoxyCTX3C [M+H]+ as a precursor ion. (Large size of full range MS/MS spectrum).
